# Gene Silencing of SOCS3 by siRNA Intranasal Delivery Inhibits Asthma Phenotype in Mice

**DOI:** 10.1371/journal.pone.0091996

**Published:** 2014-03-17

**Authors:** M Paz Zafra, Carla Mazzeo, Cristina Gámez, Ainara Rodriguez Marco, Ana de Zulueta, Veronica Sanz, Izaskun Bilbao, Jesús Ruiz-Cabello, Jose M. Zubeldia, Victoria del Pozo

**Affiliations:** 1 Department of Immunology, IIS-Fundación Jiménez Díaz, Madrid, Spain; 2 CIBER de Enfermedades Respiratorias, Madrid, Spain; 3 Advanced Imaging Unit, Centro Nacional de Investigaciones Cardiovasculares, and Universidad Complutense Madrid, Madrid, Spain; 4 Allergy Section and Experimental Medicine Unit, Gregorio Marañón Hospital, Madrid, Spain; Harvard Medical School, United States of America

## Abstract

Suppresors of cytokine signaling (SOCS) proteins regulate cytokine responses and control immune balance. Several studies have confirmed that SOCS3 is increased in asthmatic patients, and SOCS3 expression is correlated with disease severity. The objective of this study was to evaluate if delivering of SOCS3 short interfering RNA (siRNA) intranasally in lungs could be a good therapeutic approach in an asthma chronic mouse model. Our results showed that intranasal treatment with SOCS3-siRNA led to an improvement in the eosinophil count and the normalization of hyperresponsiveness to methacholine. Concomitantly, this treatment resulted in an improvement in mucus secretion, a reduction in lung collagen, which are prominent features of airway remodeling. The mechanism implies JAK/STAT and RhoA/Rho-kinase signaling pathway, because we found a decreasing in STAT3 phosphorylation status and down regulation of RhoA/Rho-kinase protein expression. These results might lead to a new therapy for the treatment of chronic asthma.

## Introduction

Asthma is a disorder of the conducting airways that leads to variable airflow obstruction in association with airway hyperresponsiveness. It is characterized by inflammatory cell infiltration, mainly of eosinophils and lymphocytes, and reversible bronchoconstriction. As the disease becomes more severe, the airway acquire an altered repair response, and structural changes cause remodeling due to the secretion of factors and cytokines that induce mucous cell metaplasia, angiogenesis, and subepithelial fibrosis [1], [2].

Cytokines are one of the main soluble molecules that participate in the initiation, perpetuation, execution, and resolution of the inflammatory response. Therefore, tight control of cytokine production and function is required to prevent an imbalance in the magnitude, duration, and remission of the immune response. SOCS proteins are recognized as an important mechanism in the negative regulation of several cytokine pathways, and more recent studies have revealed that SOCS proteins play additional roles in many immunological processes as asthma [3], [4].

The cytokine profile in asthma is typified by interleukin (IL-) 4, IL-5, and IL-13 that are typical Th2 cytokines. These cytokines bind to membrane receptors to activate complex signal transduction pathways. SOCS is a family of molecules that suppress the JAK-STAT signaling pathway and regulate Th cell differentiation. SOCS3 is an inhibitor that is relatively specific to STAT3; in addition, it can inhibit other signaling pathways such as Ras/ERK and PI3K, which affect cell proliferation, survival, and differentiation [5], [6]. Th2 cells contain high levels of SOCS3 proteins. Our group has recently described and increased SOCS3 expression in eosinophils from asthmatic patients [7].

In patients with asthma and atopic dermatitis, the expression levels of SOCS3 transcripts in T cells are closely correlated with serum IgE levels and disease severity [8], [9], [10]. Previously, transgenic expression of SOCS3 in T cells resulted in increased Th2 cell production, airway eosinophilia, and airway hyper-responsiveness (AHR) in a murine model of OVA-allergic asthma [8]. In fact, T cell treatment with SOCS3-siRNA has been demonstrated to suppress the development of allergic inflammation in another murine model of asthma [11].

Gene silencing by RNA interference, in which the expression of the target gene is silenced in a post-transcriptional manner, has arisen as a potential treatment for many diseases. The intranasal administration of naked siRNA has opened new possibilities in drug delivery and respiratory therapy [12], [13]. Therefore, we have developed a therapeutic approach in a murine model of chronic asthma by delivering SOCS3-siRNA intranasally.

## Methods

### Ethics statement

All experimental procedures were carried out in strict accordance with the international and national (Real Decreto 1201/2005) guidelines and were approved by the Fundación Jiménez Díaz Animal Research Ethics Committee. All protocols were performed under conditions to minimize animal suffering.

### Animals

Male A/J mice (specific pathogen-free, 5 weeks old) were purchased from Harlan Iberica. A total of 68 animals were included in the study. Mice were assigned to 4 experimental groups: saline solution group (SS, n = 17), ovalbumin-sensitized group (OVA, n = 16), ovalbumin-sensitized non-target siRNA- treated group (OVA siNT, n = 16), and ovalbumin-sensitized SOCS3 siRNA-treated group (OVA siSOCS3, n = 19).

### Recombinant proteins and reagents

Ovalbumin (OVA) grade V was purchased from Sigma-Aldrich (MO, USA). Antibodies against phospho-STAT3 (Ser^727^), STAT3, SOCS3, and β-actin were obtained from Cell Signaling Technology (MA, USA); anti-mouse ROCK-2 was acquired from Sta Cruz Biotech (CA, USA), and anti-mouse RhoA was purchased from Cytoskeleton (CO, USA). Flow cytometry antibodies (anti-mouse CD49d PE, CD3 PE, and CD19 FITC) were all purchased from BD (NY, USA).

### Chronic asthma model

Mice were sensitized on days 0 and 14 by intraperitoneal injection of 10 μg OVA (grade V; Sigma Aldrich, MO, USA) and 20 mg Al(OH)_3_ in PBS. Then, mice were immunized during five weeks, four times per week, starting on day 27 and finishing on day 58, by placing them in a small box, where they were later anesthetized with inhaled isofluorane (Forane, Abbot, IL, USA) and then immunized with 15 μl of OVA (1 mg/ml) or saline solution (SS) intranasal; 24 hours after the last OVA administration, mice were sacrificed. The followed protocol is further detailed by S. Miyamoto et al. [14].

### Design and delivery of siRNA *in vivo*

The Accel siRNA SMART-pool duplexed of 4 predesigned mouse SOCS3-siRNA (# 1-4) were purchased from Dharmacon (IL, USA). The siRNA sequences specific for mouse SOCS3 (#1: sense 5′-GCCUCAAUCACUUUUAUAAUU-3′, antisense 5′-PUUAUAAAAGUGAUUGAGGCUU-3′; #2: sense 5′-GUAUGAUGCUCCACUUUAAUU-3′, antisense 5′-PUUAAAGUGGAGCAUCAUACUU-3′; #3: sense 5′- CUGUUUUGAAUAAUGUUUAUU-3′, antisense 5′-PUAAACAUUAUUCAAAACAGUU-3′; #4: sense 5′-GGGGAAUCUUCAAACUUUCUU-3′, antisense 5′-PGAAAGUUUGAAGAUUCCCCUU-3′) were selected, synthesized, and annealed by the manufacturer. Additionally, a non-target siRNA scrambled duplex from Dharmacon was used (sense 5′- UGGUUUACAUGUCGACUAAU-3′, antisense 5′-PUUAGUCGACAUGUAAACCAUU-3′). SOCS3-siRNA and non-target siRNA were dissolved in RNAse-free water and then given intranasally at a volume of 15 μl, previously each animal was lightly anesthesized using isofluorane. After the first sensitization with OVA, a total of 10 doses of siRNA were administered every 3 days. To determine the effective SOCS3-siRNA dose, different concentrations were tested (2-20 μM) in mice with chronic asthma. The optimal siRNA concentration selected was 2 μM.

### Confocal microscopy

Accell Cyclophilin B siRNA rhodamine labeled was purchased from Dharmacon and instilled intranasally into the mice. Twenty-four and 48 hours after the siRNA instillation, frozen lung sections were observed by confocal microscopy (Leyca Microsystems, Weztlar, Germany) at an excitation wavelength of 547 nm.

### Determination of airway responsiveness to methacholine

Mice airway responsiveness was assessed in all animal groups 1 day before the animals were sacrificed. Four individual whole body plethysmography chambers, obtained from Buxco, were used as described previously [15]. Enhanced pause (Penh), a parameter that correlates with measurement of airway resistance, was used to perform the analysis.

### X-ray CT

In vivo CT imaging was performed on a nanoPET/CT small-animal imaging system (Bioscan, Washington, DC) equipped with a micro-focus X-ray source and a high-resolution radiation-imaging device featuring a 1024×3596 pixel photodiode array with a pixel pitch of 48 μm. The mice were intraperitoneally anesthetized with ketamine/xylacine (100 mg/10 mg per kg of body weight) and positioned in a thermoregulated (38.7°C) mouse bed with an ophthalmic gel in their eyes to prevent retinal drying.

The scan parameters used for the CT measurements were 360 projections/rotations, 55 kV (peak) 145 μA current, and a detector pixel size of 141 μm.

Acquisition and reconstruction were performed using proprietary Nucline software (Mediso; Budapest, Hungary). The effects induced by intranasal challenge with ovalbumin were observed by Hounsfield Unit density change and volume change quantified manually using Osirix software (Pixmeo, Switzerland). The appropriate CT images were preset at the lung window setting (-400 center, 1240 HU width).

### Fluorescence molecular tomography (FMT)

A tail vein injection of 2 nmol of ProSense 680 agent (Perkin Elmer, MA, US) resuspended in 150 μl of saline was performed in all mice at day 58, 4 hours after the final intranasal administration of OVA. For this application, the mice were anesthetized with 2% isofluorane. Twenty hours after the fluorescence agent delivery, all mice were imaged using an FMT 1500 fluorescence tomography system (Perkin Elmer) after depilation to minimize interference with the fluorescence signal. Immediately before imaging, the mice were anesthetised using an intraperitoneal injection of ketamine/xylazine (100 μl per 10 g of animal) and positioned carefully in the FMT chamber with ophthalmic gel in their eyes to prevent retinal drying. The resulting 3D data were reconstructed and the region of interest (ROI) was defined within the chest area using TrueQuant 3D software to obtain quantifications. The same threshold was applied to all animals.

### Bronchoalveolar lavage (BAL), cell analysis

Twenty-four hours after the last administration of the antigen, the mice were anesthetized and lung lavages were performed 3 times with 0.5 ml of sterile PBS. The cells obtained were counted in a Neubauer chamber and used for cytometric analysis in a FACS CANTO II cytometer (BD, NY, USA).

### Tissue processing and histological analysis

The right lung of each mouse was extracted while the animal was under terminal anaesthesia; the lung was then immersed in 4% paraformaldehyde. Five-micrometer sections were stained with H&E to assess general morphology. Mucous-secreting goblet cells were visualized on periodic acid-Schiff (PAS), and Masson Trichrome stain was used to evaluate for subepithelial fibrosis. Cell infiltration in the tissue specimens was assessed by counting the number of cells through a semiautomatic method and Image J software was used for imaging analysis. These analyses were performed in a blind fashion, and the slides were presented in random order for each examination.

### Immunoblot

Protein extracts from mice lungs (20 μg of total protein) were resolved on SDS-PAGE and probed with specific Abs at the appropriate dilution (phospho-STAT3 1∶500, STAT3 1∶1000, SOCS3 1∶500, RhoA 1∶500, ROCK-2 1∶1000, β-Actin 1∶2000). Chemiluminescent protein bands were detected using an ECL detection system (Amersham Biosciences, GE Healthcare, Buckinghamshire, UK) according to the manufacturer's protocol.

### RNA isolation, RT-PCR, and TaqMan gene expression assays

Mice lungs were homogenized previously and then total RNA was isolated following the Trizol reagent protocol (Invitrogen, CA, USA). RNA was measured by spectrophotometry, and 1 μg of RNA was reverse-transcribed to cDNA using a high-capacity cDNA Reverse Transcription Kit (Applied Biosystems, Warrington, UK).

Quantitative real-time PCR was performed on a 7500 Real-Time PCR system (Applied Biosystems). TaqMan gene expression master mix and TaqMan gene expression assay probes (SOCS3, SOCS1, IL-13, IL-4, IL-5, IFNγ, IL-10, and IL-17A) were obtained from Applied Biosystems and were used for qRT-PCR to determine mRNA levels. Messenger RNA was calculated for each sample using the cycle threshold (C_t_) value. The relative gene expression was calculated as follows: 2-ΔΔCt, where ΔΔC_t_ = ΔC _target gene_−ΔC _actin_ [16].

### RNA quality control and miRNA microarray analysis

Total RNA was isolated from the lungs using an RNeasy Mini Kit (Qiagen, Hilden, Germany) according to the manufacturer's protocol. The integrity and quantification of each total RNA extract was assessed with an Agilent 2100 Bioanalyzer (Agilent Technologies, CA, USA) and unqualified samples were rejected. A total of 5 μg of purified RNA samples was submitted to the Gene Expression Department of Complutense University (Madrid, Spain). In total, 16 samples were analyzed: 4 from the OVA group, 4 from the OVA siNT group, 4 from the OVA siSOCS3 group, and 4 from the SS group. Following all appropriate protocols and procedures for quality control, labeling, and fragmentation of total RNA, the biotin-labeled cRNA samples were hybridized to GeneChip miRNA 3.0 Array (Affimetrix, CA, USA) according to manufacturer protocols.

The microarray normalization process was determined with the software GeneChip Expression Console (Affimetrix). Data were standardized using the RMA+DABG algorithm. Functional analysis was carried out using the Babelomics suite (http://www.babelomics.org/). Differential gene expression was conducted using the Limma package from Bioconductor (http://www.bioconductor.org/). To account for multiple testing effects, *P* values were corrected using the false discovery rate. Differentially detected signals were accepted as true when ratios of the *P* value were less than 0.01. Results were finally given in terms of “fold induction.”

### Determination of total serum IgE and OVA-specific IgE, IgG1, and IgG2A

At the time of sacrifice, blood samples were collected by cardiac puncture, and the sera were stored at −80°C until use. Total serum IgE and OVA-specific IgE, IgG1, and IgG2A were measured by ELISA (BD Biosciences/BD Pharmingen, NJ, USA).

## Results

### Localization of siRNA into lung following single intranasal administration in mice

**Figure 1:**
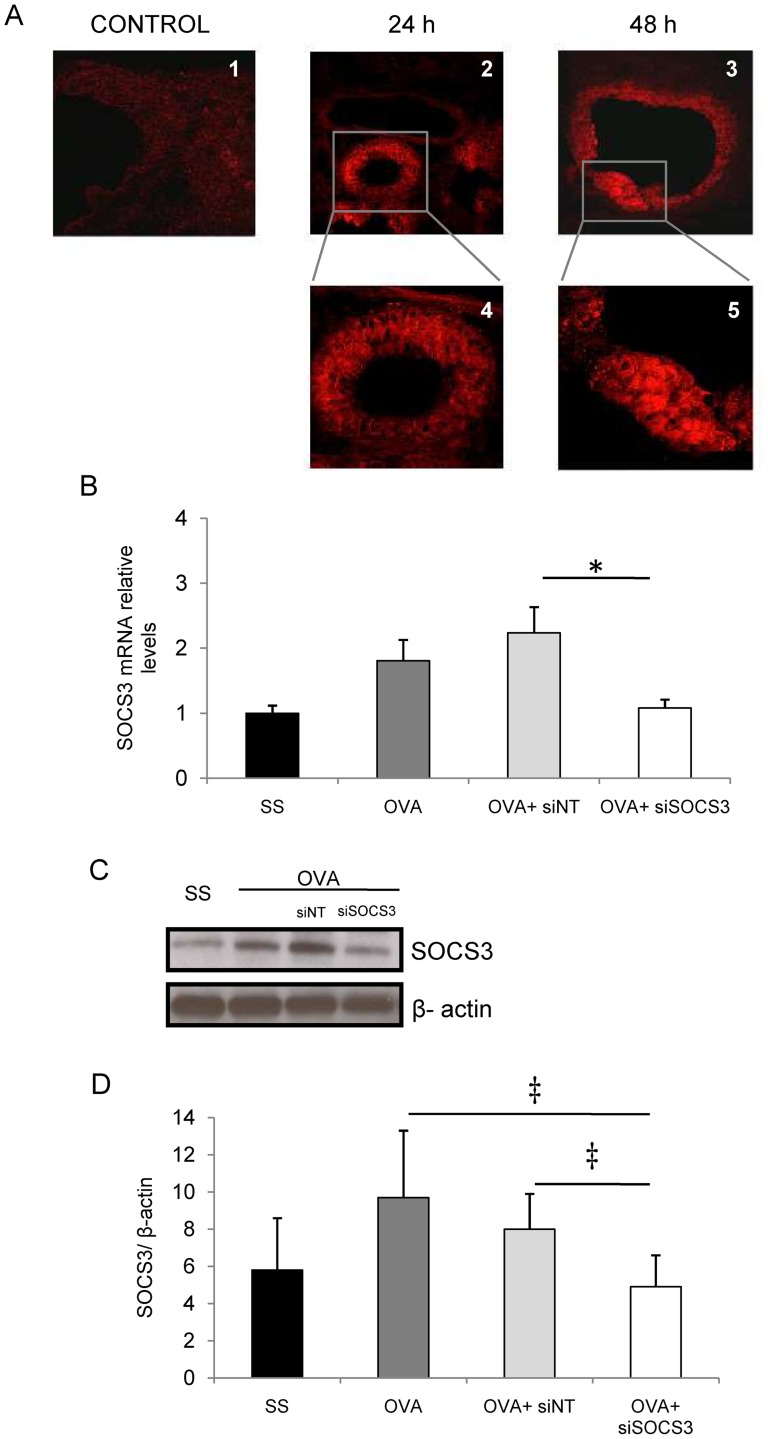
SOCS3 gene silencing in lung. **A**. Red-labeled siRNA localization in mouse lung after intranasal delivery determined by confocal microscopy. 2 μM of siRNA was administered. 1: control mouse lung after 48 h after administration of distilled water. 2 and 4: Lung airway images after 24 h from siRNA delivery. 3 and 5: Lung airways 48 h after siRNA administration. **B**. Relative SOCS3 mRNA levels were determined by real-time quantitative PCR. The results show relative gene expressions as determined by the ΔΔC_T_ method. **C**. SOCS3 Western blot analysis from lungs was achieved using an antibody against SOCS3 and β-actin. Lane 1: lung lysate from the SS group; lane 2: lung lysate from the OVA group; Lane 3: lung lysate from the OVA+siNT group; and lane 4: lung lysate from the OVA+siSOCS3 group. β-actin was used as a loading control. The picture is a representative example of several mice, all displaying similar results. **D**. SOCS3 bands were quantified by densitometry. SS group (mean ±SD, n = 13); OVA group (mean ±SD, n = 12); OVA siNT-treated group (mean ±SD, n = 12); OVA siSOCS3-treated group (mean ±SD, n = 15). ‡*P*<0.001 and *P<0.05 between groups.

Firstly, we performed an assay to determine whether naked siRNA delivered intranasally reached the lung intact and was not degraded by RNAses in lung airways. After instillation of control siRNA labeled (2 μM) with a fluorophore (Rhodamine) in the airways, red fluorescence was observed by confocal microscopy in response to the 547 nm laser beam. As shown in Figure 1A, strong red fluorescence was localized in the lungs, predominantly in the peribronchial epithelial cells either 24 or 48 hours after administration. No fluorescence was observed in the lungs of the control group.

### SOCS3 evaluation after instillation in a chronic asthma model

We set out to study SOCS3 gene expression and encoded protein in lung tissue by quantitative PCR and Western blotting after SOCS3-siRNA therapy. The plot in Figure 1B shows how SOCS3 gene expression was inhibited in the lungs of mice that received SOCS3-siRNA therapy (OVA siNT vs OVA siSOCS3, *P*<0.05).

The immunoblotting analysis confirmed these results (Fig. 1C). Thus, both the group treated with SOCS3-siRNA and the control group showed similar SOCS3 protein expression (47 vs 58 arbitrary units; *P*>0.05), i.e. a strong inhibition relative to the OVA and OVA siNT groups *(P*<0.001 and *P*<0.05, respectively; Fig. 1D).

To assess whether the SOCS3-siRNA delivery had caused an alteration in other important regulators belonging to the same family, new measures of SOCS1 mRNA relative levels revealed no change in this gene expression (see Figure S1).

Finally, to further analyze whether the silence therapy had affected other organs, SOCS3 quantitative PCR was performed in liver and spleen from all mice and we observed that SOCS3-siRNA treatment did not alter SOCS3 gene expression in tested organs (see Figures S2 and S3).

### Reduced inflammation and airway hyperresponsiveness after SOCS3 silencing

**Figure 2:**
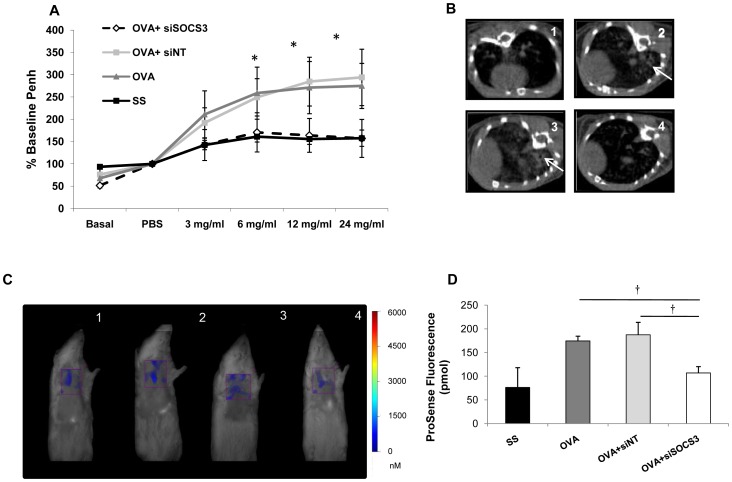
*In vivo* techniques to assess the effect of SOCS3 gene silencing in lungs. **A**. Airway hyperresponsiveness to methacholine challenge. Methacholine dose response curve 24 hours after last OVA administration. Saline group (mean ±SD, n = 13); OVA group (mean ±SD, n = 12); OVA siNT-treated group (mean ±SD, n = 12); and OVA siSOCS3-treated group (mean ±SD, n = 15). **B**. Native axial micro-CT images of the lungs of typical mice from each experimental group, where hyperintense areas (arrow) correspond to tissue with more fluid than normal. 1: SS n = 4; 2: OVA n = 4; 3: OVA siNT n = 4; 4: OVA siSOCS3 n = 4. *P<0,05 between groups. **C**. In vivo FMT tomographic imaging. Lung fluorescence after ProSense 680 administration in mice. Three-dimensional regions of interest were placed within the lung region. 1: SS group; 2: OVA group; 3: OVA siNT group; 4: OVA siSOCS3 group. **C**. 3D FMT quantification of pulmonary inflammation, expressed as total pmoles per lung ± S.D (†*P*<0,01) of ProSense 680 fluorescence within the lung region of each mouse from all the study groups (SS n = 4, OVA n = 4, OVA siNT n = 4, OVA siSOCS3 n = 4).

Airway function was assessed using whole body plethysmography 24 hours before sacrifice. OVA and OVA siNT-treated mice developed a significantly enhanced response to methacholine when compared to saline animals (Fig. 2A). As expected, SOCS3 siRNA-treated mice displayed significantly inhibitory responses to cholinergic stimulation with different methacholine doses (6, 12, and 24 mg/ml) when compared to the other asthmatic groups. Therefore, SOCS3-siRNA therapy protected against methacholine-induced AHR.

Typical micro-CT images from each group are illustrated in Figure 2B. There is an increase in density units in animal lungs from the OVA and OVA siNT-treated groups (478±271 HU), as can be observed in the images (Fig 2B, panels 2 and 3). In mice treated with SOCS3-siRNA, a 2.7-fold reduction in HU was obtained (178.75±60.813 HU), although these differences did not reach statistical significance (Fig 2B, panel 4).

3D FMT imaging in multiple animals (Fig. 2C) displays a similar pattern and extension of fluorescence signal in OVA and OVA siNT-treated mice. Similarly, control and SOCS3 siRNA-treated mice show a decrease in fluorophore concentration (ProSense 680 nm). The concentrations derived from FMT (Fig. 2D) performed in asthmatic (OVA and OVA siNT) mice were 106±13 pmol/lung in transient SOCS3 knock-down versus 174±10 and 187±26.2 pmol/lung, respectively; *P*<0.05.

**Figure 3:**
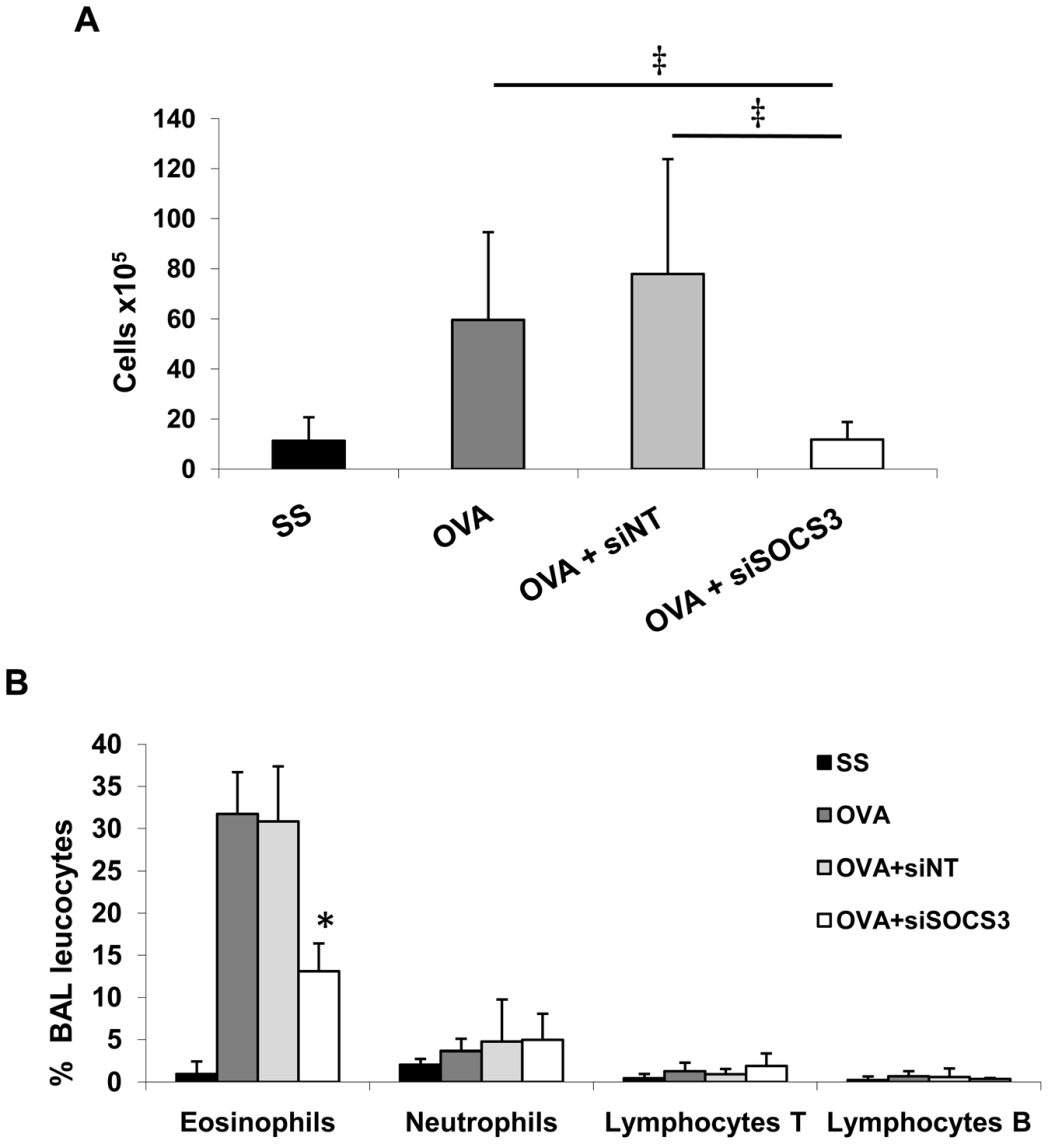
Analysis of cell populations in BAL. **A**. Total cell counts in BAL. **B**. Leukocyte population analysis by flow cytometry in BAL. SS group (mean ±SD, n = 13); OVA group (mean ±SD, n = 12); OVA siNT-treated group (mean ±SD, n = 12); and OVA siSOCS3-treated group (mean ±SD, n = 15) ‡*P*<0.001 and *P<0.05 between groups.

Cellular airway inflammation is a pivotal event in OVA-induced airway sensitization and, as expected, the SOCS3-siRNA treatment produced changes in the total number and composition of cells from the BAL. Figure 3A shows that after SOCS3-siRNA therapy, the absolute number of BAL cells significantly decreased in comparison to the OVA group (11.8±7.1 10^5^ cells versus 59.6±35.1 10^5^ cells, *P*<0.001).

Flow cytometry was used to evaluate the percentage of different cell subpopulations in BAL. As shown in Fig. 3B, treatment with SOCS3-siRNA altered the cellular profile, resulting in a significant reduction in eosinophils (a 41% decrease) in BAL fluid *(P*<0.05). We did not find any significant changes in other leukocyte populations measured, such as neutrophils or lymphocytes T and B. These results indicate that administration of SOCS3-siRNA into the lung modulates allergen-induced BALF eosinophilia.

### Histopathologic examination

**Figure 4:**
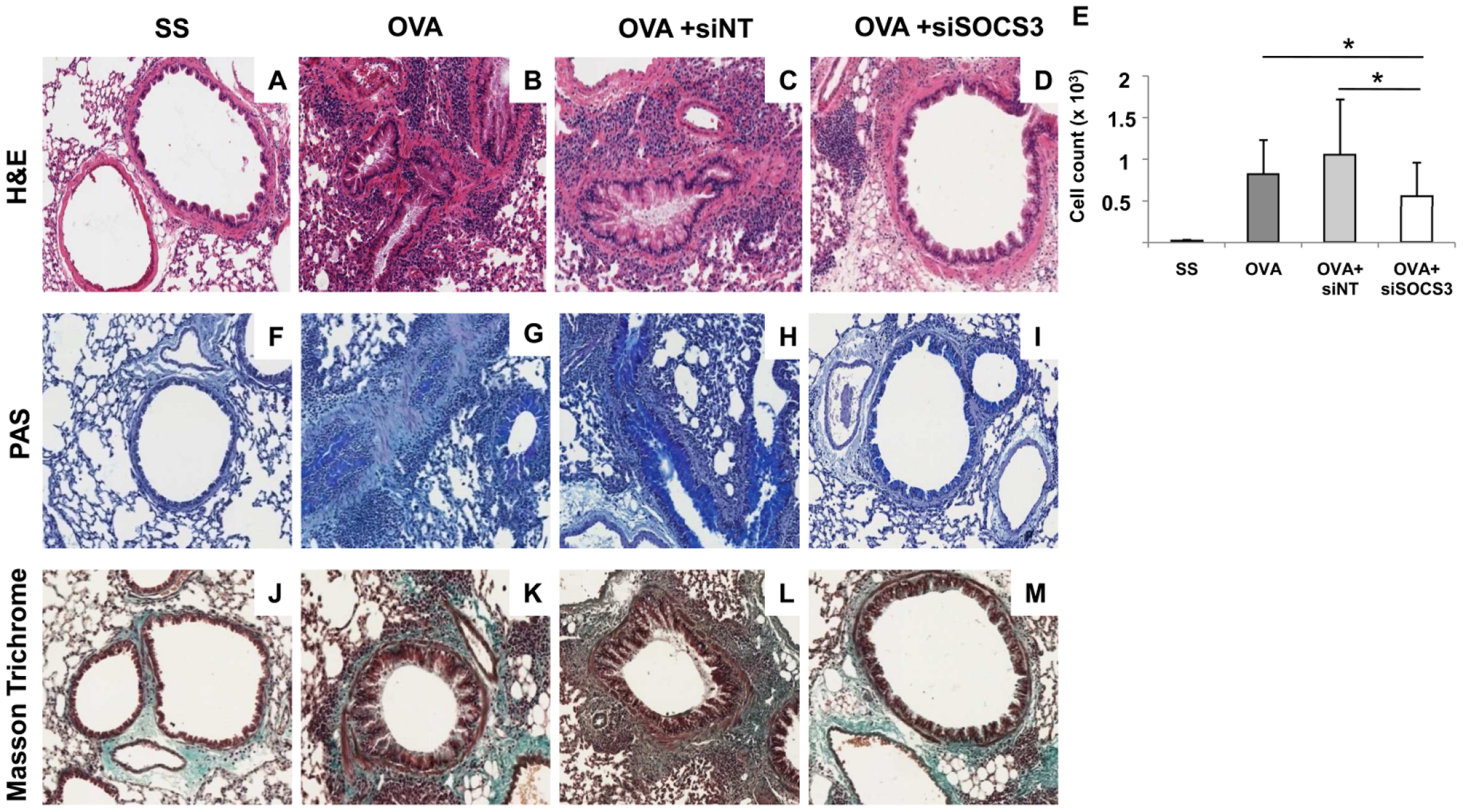
Pulmonary histopathologic findings in chronic asthma: effect of SOCS3-siRNA therapy. Comparison of structural changes in airways of negative control (SS, n = 13), untreated positive control (OVA, n = 12), non-target siRNA-treated (siNT, n = 12), and SOCS3 siRNA-treated (SOCS3-siRNA, n = 15) mice shown with H&E (row A–D), PAS (row F–I) and Masson Trichrome (row J–M) staining of lung sections, in at least 3 independent experiments. Representative photomicrographs of excised pulmonary tissue from the saline group are in the first column, the second column presents the OVA group, the third depicts the OVA group, and the SOCS3- siRNA group is represented in the fourth column. **E**. Cell infiltration in the specimens. Saline group (mean ±SD, n = 13); OVA group (mean ±SD, n = 12); OVA siNT-treated group (mean ±SD, n = 12); and OVA siSOCS3-treated group (mean ±SD, n = 15). *P<0.05 between groups.

SOCS3-siRNA treatment resulted in a reduction of inflammatory infiltrates in the perivascular and peribronchial regions in mice lungs (Fig. 4D) compared to OVA, and OVA siNT groups (Fig. 4B and C). These infiltrates were quantified by semiautomatic analysis and the results are presented in Figure 4E.

### SOCS3-siRNA therapy abrogates mucous presence and reduces collagen deposits in the airways

Excessive mucous secretion from hyperplasic goblet cells is also a characteristic feature of the asthmatic airway. To determine if the extent of mucous cell metaplasia following SOCS3 knockdown is modified, paraffin-embedded sections of lung were stained with PAS that stains mucous-producing goblet cells in asthmatic airways. In mice with chronic asthma as well as in those treated with the non-target siRNA (Fig. 4G and H), an increase in PAS-positive cells was observed in the bronchial epithelium when compared with saline controls (Fig. 4F). These positive cells almost disappeared in OVA mice that had received SOCS3-siRNA therapy and therefore displayed a similar pattern to that observed in the airways of mice from saline group (Fig. 4I).

Increased collagen deposition is a hallmark of airway remodelling due to prolonged inflammation with chronic asthma. Connective tissue was examined in lung sections stained with Masson Trichrome. Saline mice presented a thin uniform layer of matrix in the peribronchial subepithelial region (Fig. 4J), whereas in chronic asthmatic mice we detected an increase in matrix thickness in the subepithelial layer of the airways as well as in the perivascular regions (Fig. 4K), as shown by the increase in the extent of collagen deposition and intensity of staining. Similar results were obtained for OVA mice treated with the non-target siRNA (Fig. 4L). When asthmatic mice were treated intranasally with SOCS3-siRNA, we found that matrix deposition was consistently abrogated in the same regions (Fig. 4M).

### Humoral response was not altered in chronic asthmatic mice after SOCS3 silencing

We also tried to ascertain whether SOCS3-siRNA treatment might alter the humoral immune response. As can be seen in Table 1, determination of serum IgE revealed that after SOCS3-siRNA treatment, total IgE levels were not altered compared with mice with chronic asthma (OVA group). Specific IgG2a and IgG1 measurements against OVA indicated that the response against the Ag was not modified in the SOCS3 silencing group. In this group, however, a slight reduction in OVA-specific IgE level was observed.

**Table 1:** Total and specific Ig in serum.

Group	Specific OVA Ig (O.D)*			
	IgG1	IgG2	IgE	Total IgE (ng/ml)
**SS**	0.05±0.04†	0.05±0.03†	0.06±0.02†	60.2±67.1†
**OVA**	0.41±0.16	0.34±0.22	0.12±0.1	130±25.6
**OVA + siRNA NT**	0.49±0.24	0.26±0.24	0.18±0.13	156.1±58.6
**OVA + siRNA SOCS3**	0.51±0.28	0.32±0.28	0.08±0.07	150.3±3.3

*Ig levels values represented (mean ± SD) for each group (12–17 mice/group).

† *P*<0.05 vs other groups.

### Effect of SOCS3 down-regulation on quantitative expression of cytokine genes in lungs

**Figure 5:**
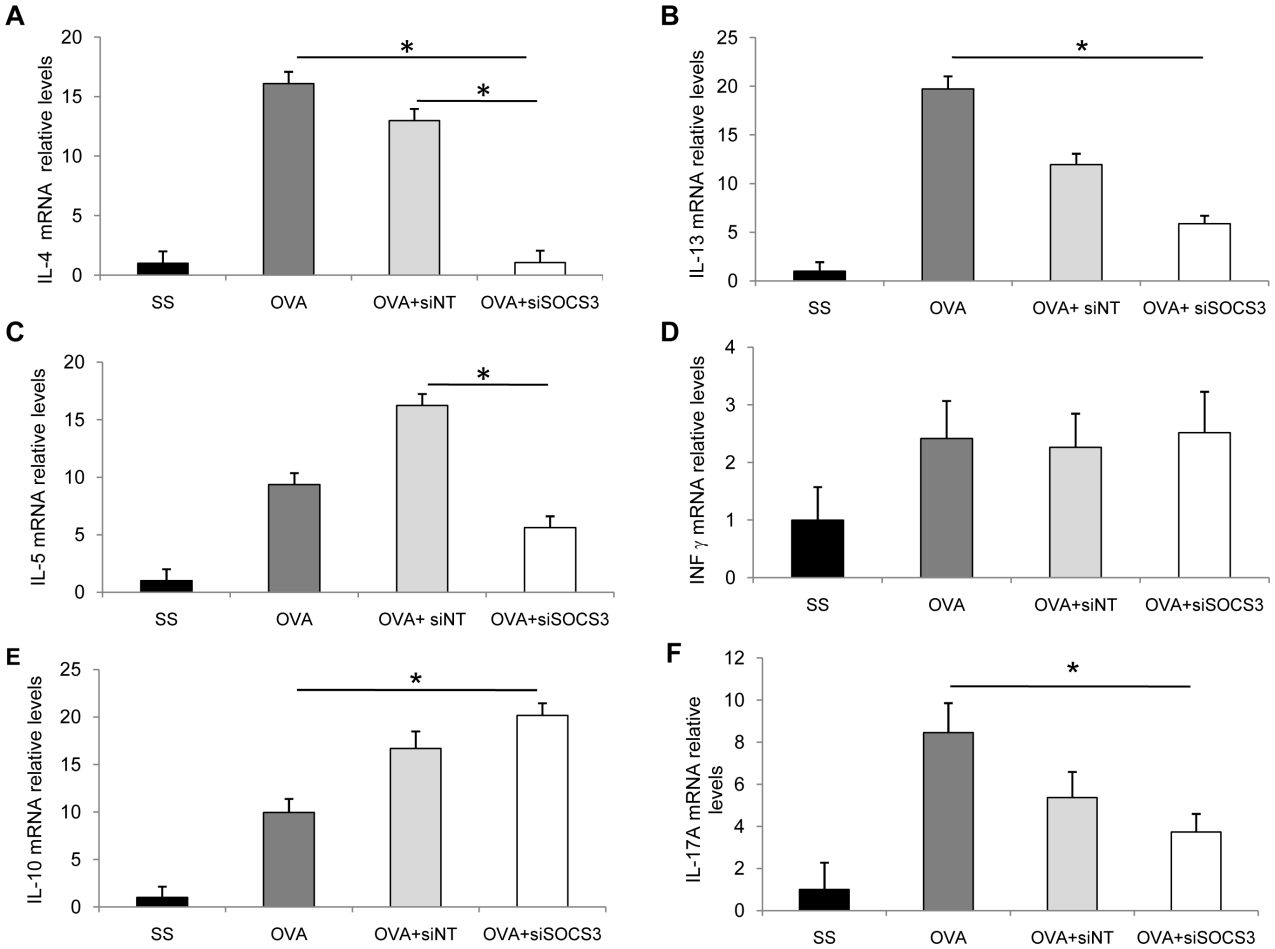
Effect of SOCS3-siRNA therapy on quantitative expression of cytokine genes in lungs. mRNA levels of IL-5 (A), IL-4 (B), IL-13 (C), INFγ (D), IL-10 (E), and IL-17A in lungs of different groups of mice, measured by real-time quantitative PCR. The results show relative gene expression as determined by the ΔΔC_t_ method. SS group (mean ±SD, n = 13); OVA group (mean ±SD, n = 12); OVA siNT- treated group (mean ±SD, n = 12); and OVA siSOCS3-treated group (mean ±SD, n = 15). Significant differences for IL-5 and IL-4 (P<0.05) expression levels were obtained for OVA SOCS3-siRNA vs OVA and OVA siNT groups. For IL-13, IL-10, and IL-17A, significant differences were achieved only between the OVA and OVA SOCS3-siRNA groups (*P<0.05 between groups).

We assessed the effect of SOCS3 silencing on lung cytokine levels. The results indicated that Th2 cytokines (IL-4, IL-13, and IL-5) were increased in mice with chronic-induced asthma (*P*<0.05, Figure 5 A, B, C) when compared with saline mice. When SOCS3-siRNA treatment was delivered, the mRNA levels of IL-4, IL-5, and IL-13 were significantly lower than in the OVA group (Fig. 5A, B and C, *P*<0.05). We also found elevation of INFγ mRNA expression levels in asthmatic mice groups, but no significant changes were found in the levels of INFγ after SOCS3 silencing (Figure 5D).

We also evaluated the regulatory axis (IL-10 and IL-17A gene expression). A marked increased in IL-10 mRNA expression, as compared with OVA mice, was achieved in mice with SOCS3 down-regulation, as shown in Figure 5E (10-fold, *P*<0.05). We further corroborate IL-10 increased production after SOCS3 down-regulation in BAL fluids (Data not shown). By contrast, IL-17A mRNA levels were augmented in OVA mice and inhibited in SOCS3 siRNA-treated mice (Fig. 5F, *P*<0.05).

In general, mice treated with the non-target siRNA displayed a similar cytokine expression profile than OVA mice; although the non-specific siRNA treatment seems to affect IL-13, IL-10 and IL-17A mRNA relative levels, these alterations do not reach significant differences.

Thus, analysis of the expression levels of several cytokines in the study groups showed that IL-5, IL-13, IL-4, and IL-17A were the predominant mediators down-regulated after SOCS3 silencing; by contrast, IL-10 is up-regulated in the local inflammatory process in airways.

### Signaling pathways

**Figure 6:**
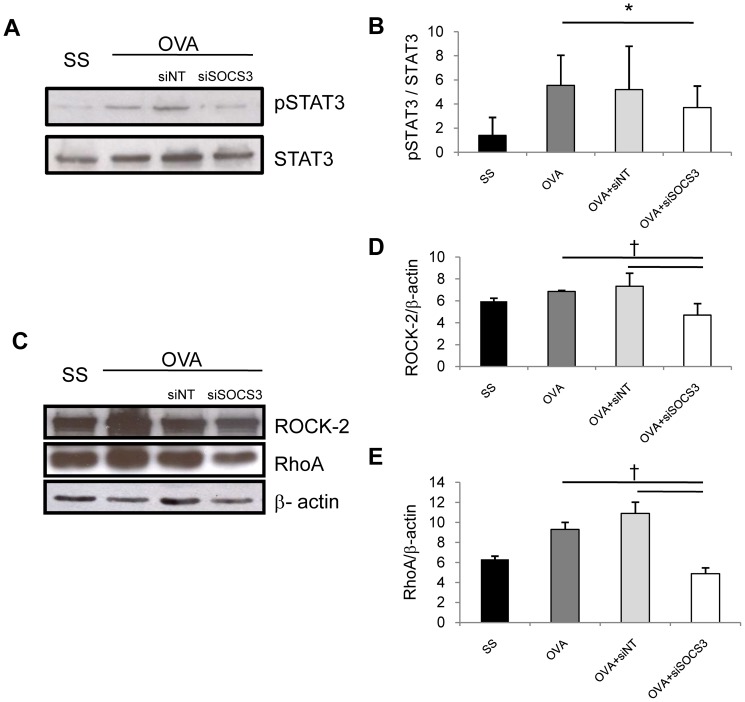
Signaling routes modified after SOCS3-siRNA therapy. Total lung proteins from mice for all the study groups were isolated and Western blot analyses were performed to measure p-STAT3, RhoA, and ROCK-2 activation. pSTAT3 expression was measured against total STAT3 protein (A) and densitometric quantified (B). ROCK-2 and RhoA expression were detected and normalized against β-actin protein (C). Band densitometries were performed to quantified protein of ROCK-2 (D) and RhoA (E). SS group (mean ±SD, n = 13); OVA group (mean ±SD, n = 12); OVA siNT-treated group (mean ±SD, n = 12); and OVA siSOCS3-treated group (mean ±SD, n = 15). †*P*<0.01 between groups and *P<0.05 between groups.

The JAK-STAT pathway is activated in chronic asthma. To ascertain STAT3 phosphorylation state after SOCS3-siRNA delivery, we performed a Western blot analysis. STAT3 phosphorylation is induced in OVA and OVA siNT-treated mice, while mice treated with SOCS3-siRNA showed a reduction in pSTAT3 status (Fig. 6A). Figure 6B summarizes the pSTAT3 protein expression quantified by densitometry and normalized with STAT3 levels.

The RhoA/Rho kinase pathway is directly implicated in asthma, promoting bronchoconstriction, airway remodeling, and airway inflammation. In our model, RhoA and ROCK-2 protein expressions were increased in chronic asthmatic mice (OVA and OVA siNT groups). Whereas in mice with SOCS3-siRNA treatment, we found less protein expression of both intermediates: ROCK-2 and RhoA (Fig. 6C). Protein bands were quantified by densitometry; the results are plotted in Figure 6D and 6E, respectively.

### MicroRNAs profiling after SOCS3 transient knockdown

Because miRNAs are now recognized as key regulatory elements in gene expression, we determined which miRNAs had modified their expression following SOCS3-siRNA therapy in the asthma mouse model using microarray analysis. Table 2 displays all the miRNAs affected after SOCS3-siRNA therapy. The comparison of OVA and OVA siNT versus the OVA siSOCS3 group shows a total of 25 miRNAs that are down-regulated in the asthmatic treated groups (first column), while 11 miRNAs were augmented due to the treatment (second column).

**Table 2:** List of significantly modulated miRNAs after SOCS3 siRNA therapy compared to the non-treated asthmatic groups (*P*<0.05), and their respective fold induction (FI).

Downregulated*		Upregulated†	
miRNA	FI	miRNA	FI
miR-17	2,153	miR-466g	0,448
miR-674	2,032	miR-1935	0,459
miR-25	2,069	miR-1247	0,269
miR-195	2,917	miR-705	0,431
miR-497	2,034	miR-5107	0,467
miR-199b	2,4	miR-3104	0,467
miR- 146b	2,527	miR-1224	0,481
miR -1839	2,234	miR-130a	0,376
miR-122	3,099	miR-1949	0,422
miR -106b	2,655	miR -200b	0,494
miR-152	2,589	miR-1247	0,464
miR-10a	3,042		
miR-199a	5,802		
miR-200a	2,105		
miR 30B	2,81		
miR-199a1	2,948		
miR-126	2,26		
miR-182	2,2		
miR-184	2,365		
miR -181c	3,317		
miR let-7d-star	2,74		
miR-1964	2,112		
miR- 665	2,682		
mir-3098	2,252		
miR-671	2,136		

*miRNAs downregulated in the siRNA SOCS3-treated group.

† miRNAs upregulated in the siRNA SOCS3-treated group.

## Discussion

The present study provides new insights in asthma therapy. We have developed and successfully tested an intranasally delivered siRNA therapy in an OVA-induced mouse model of chronic asthma. Transient knockdown of SOCS3 by naked siRNA in chronic asthmatic mice has led to a decrease in lung eosinophilia, as well as a significant reduction of AHR and mucous in the airways, therefore improving chronicity and remodelling.

The inhibition of airway alteration is measured as a reduction in cellular infiltration, globet cell hyperplasia, and matrix deposition. A histologic analysis of the airways after SOCS3 silencing revealed a diminished perialveolar and peribronchial cellular infiltration and a conserved structure in the airway epithelium.

In our model of asthma SOCS3-siRNA treatment produce also a decreased expression in IL-5, IL-13, and IL-4. This indicates that the therapy directly regulates the Th2 differentiation rate. It is well known that IL-5 actively participates in promoting eosinophil infiltration to the airways, so it is not surprising that after silence therapy IL-5 gene expression in lungs mice was reduced and this correlated with a decreased of eosinophil numbers.

After chronic OVA challenge, IL-4 mRNA levels are dramatically up-regulated in the lungs, as well as in splenocyte cultures stimulated with the OVA antigen (see Figure S4). Once in the airways, IL-4 has the capacity to induce IgE isotype switching and increase mucous production by airway epithelial cells. Moreover, IL-4 is one of the key cytokine regulating the differentiation to the Th2 phenotype. Similarly, we have found high levels of IL-13 in the airways of asthmatic mice. This IL has been traditionally related to an increase in AHR [17]. There have been many efforts to find inhibitors of these 2 cytokines, but the outcomes have yet to fulfill expectations. Kasaian et al. has recently published a therapy in which a dual IL-4/IL-13 antagonist reduces lung inflammation, AHR and IgE production in an OVA-induced asthma murine model [18]. Interestingly, SOCS3 knockdown down-regulated IL-4 and IL-13 in our experimental model, thus leading to an improvement in AHR parameters and reducing specific IgE production.

Another novel finding in this work is the reduction observed in the RhoA/Rho-kinase signaling pathway after SOCS3 silencing. In animal models of allergic bronchial asthma it has been shown that RhoA mediates the augmentation of bronchial smooth muscle contractility, which is one of the causes of AHR. Recently, ROCK has been involved in the regulation of allergic inflammation [19]. In fact, ROCK inhibitors have been tested as potential treatments for AHR in asthma [20]. Moreover, it has been described that IL-13 as well as IL-4 augment bronchial smooth muscle contractility with an up-regulation of RhoA protein, in the case of IL-4 probably through STAT6 activation [21]–[24]. With our treatment, we have reduced both cytokines and thus we have controlled the smooth muscle activation and therefore AHR.

It is known that eosinophils are a potential source of IL-17 within asthmatic airways [25], and although IL-23 and IL-17 cells induce Th17-cell-mediated neutrophilic airway inflammation, it has also been reported that cells producing these interleukins also up-regulate Th2 cell-mediated eosinophilic airway inflammation in mice [26]. According to this data, we have detected elevated IL-17A levels in asthmatic mice lungs; these levels reverted with the SOCS3 silencing treatment. At the same time, an increase in IL-10 expression in lungs was achieved in SOCS3-siRNA treated group. These results clearly point to SOCS3 as a modulator of the IL-10/IL-17 regulatory axis.

SOCS3 deficiency in T helper cells resulted in constitutive STAT3 activation, which in turn enhances TGFβ1 and IL-10 production [27]. In our model we have reduced STAT3 phosphorylation after SOCS3 silencing and elevated IL-10 production. This reduced phosphorylation is probably due to we did not abrogate SOCS3 levels completely. In this way, our therapy is focused on resetting the natural regulation process executed by the suppressor to restore the normal phosphorylation levels of the intermediates, in this case STAT3. Supporting this idea, there is a study that states that local inhibition of the STAT-3/STAT-5/SOCS3 dependent feedback loop has been shown to suppress experimental allergic asthma [28].

The major advantage of using naked siRNA is simplicity, as there is no longer cause for concern about the toxicity and inflammatory responses associated with certain delivery vectors. In addition, given intranasally, siRNA is a non-invasive and natural means of delivering therapeutic agents into the lungs. Naked siRNA targeting has been shown to be successful in inhibiting viral lung infection in mouse [29] and rhesus macaque [30]. In fact, a human clinical trial currently in phase II is using this route to deliver siRNA for the treatment of human RSV infection [31].

We have previously demonstrated that eosinophils are one of the major SOCS3-producing cells (7). We have also observed a correlation between BAL eosinophils and SOCS3 expression (r = 0.97, *P*<0.05). Targeting SOCS3 in the airways, where there is a massive eosinophil presence, may be an important mechanism of eosinophil reduction by inhibiting their recruitment to the airways.

MicroRNA-driven RNA interference is a newly recognized and evolutionary conserved regulatory mechanism [32]. We have used microarray analysis to determine miRNAs expression profiles in our chronic asthmatic mice after SOCS3-siRNA therapy. miRNA modulated by transient SOCS3 knock-down provide information about regulations operated at a postranscriptional level. Moreover, 2 of the miRNA that appeared downregulated after the SOCS3-siRNA therapy (miR-146b and 126) have been previously involved in asthma disease [32], [33]. It would be worthwhile to conduct further studies of the mechanisms involved in the regulation of those miRNAs and others we have found.

Using fluorescence molecular tomography (FMT), we have obtained visual discrimination, measurement, and quantification of asthma progression and have seen how our therapeutic response has evolved in vivo. Quantification of the cathepsin-activated ProSense 680 signal provides a noninvasive measure of pulmonary eosinophilia due to specific protease activation of ProSense by eosinophils [34]. Moreover, FMT results have been further corroborated by conventional, invasive techniques and ex vivo measurements. In addition, X-ray computed tomography has provided structural and anatomical lung information about the chronicity in asthma, showing higher density regions in lungs where the inflammatory process is taking place. These brighter areas associated with fluid accumulation were reduced after SOCS3-siRNA therapy. All together, these 2 new techniques allow us to non-invasively and longitudinally visualize and quantify inflammation in the lung and monitor therapeutic efficacy in vivo.

The advantages of our therapy are that it is easier to administer and it is delivered locally unlike in the Moriwaki et al. study [11], in which SOCS3 was downregulated in T cells *in vitro* and then adoptively transferred to mice). Furthermore, as in T cells and eosinophils, other cells in the airways are probably over-expressed SOCS3, and our therapy allows the siRNA to be taken up by other cells in the airways, thus increasing their effectiveness. These two different approaches have lead to different results. Thankfully to the local therapy, the siRNA has the ability to down regulate SOCS3 in the whole lung environment; thus, we have obtained reduced levels of IL17A mRNA and in turn, increased expression of IL-10. However, the T cell treatment with SOCS3 siRNA reported by Moriwaki et al. produced increased IL17 levels in BAL fluids.

In conclusion, we have demonstrated using different in vitro and in vivo techniques that SOCS3-siRNA intranasal delivered in a chronic asthma mouse model leads to inhibited asthmatic responses. These results may hold therapeutic potential for asthma patients.

## Supporting Information

Figure S1 **SOCS1 mRNA relative levels are not altered by local SOCS3-siRNA treatment in lungs.**
(TIF)

Figure S2 **SOCS3 gene expression in liver after intranasal SOCS3-siRNA delivering.**
(TIF)

Figure S3 **SOCS3 gene expression in spleen after intranasal SOCS3-siRNA delivering.**
(TIF)

Figure S4 **OVA treatment up regulates IL-4 expression in culture splenocytes from mice previously OVA immunized.**
(TIF)
