# Correction: Gene Silencing of SOCS3 by siRNA Intranasal Delivery Inhibits Asthma Phenotype in Mice

**DOI:** 10.1371/journal.pone.0105924

**Published:** 2014-08-14

**Authors:** The PLOS ONE Staff

The second affiliation for the ninth author is not indicated. Jose M. Zubeldia is affiliated with: Allergy Section and Experimental Medicine Unit, Gregorio Marañón Hospital, Madrid, Spain, and CIBER de Enfermedades Raras (CIBERER-U761), Madrid, Spain.
