# Supplementary figures and images for: Gene Silencing of SOCS3 by siRNA Intranasal Delivery Inhibits Asthma Phenotype in Mice
Source: PLoS One. 2014 Mar 17;9(3):e91996. doi: 10.1371/journal.pone.0091996 (PMC3956882; doi:10.1371/journal.pone.0091996)

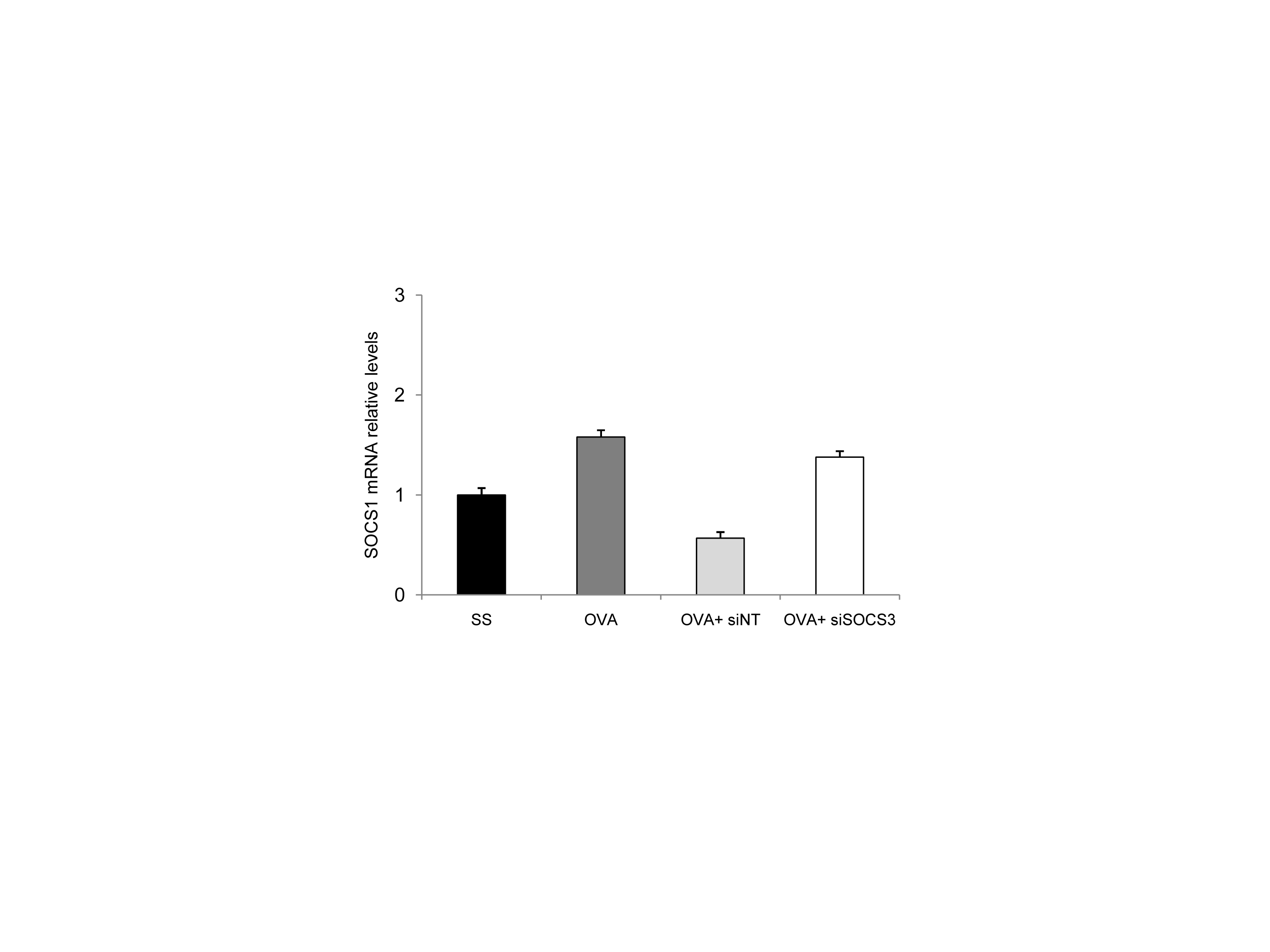

Supplement: Figure S1 — SOCS1 mRNA relative levels are not altered by local SOCS3-siRNA treatment in lungs. (TIF) [file pone.0091996.s001.tif]

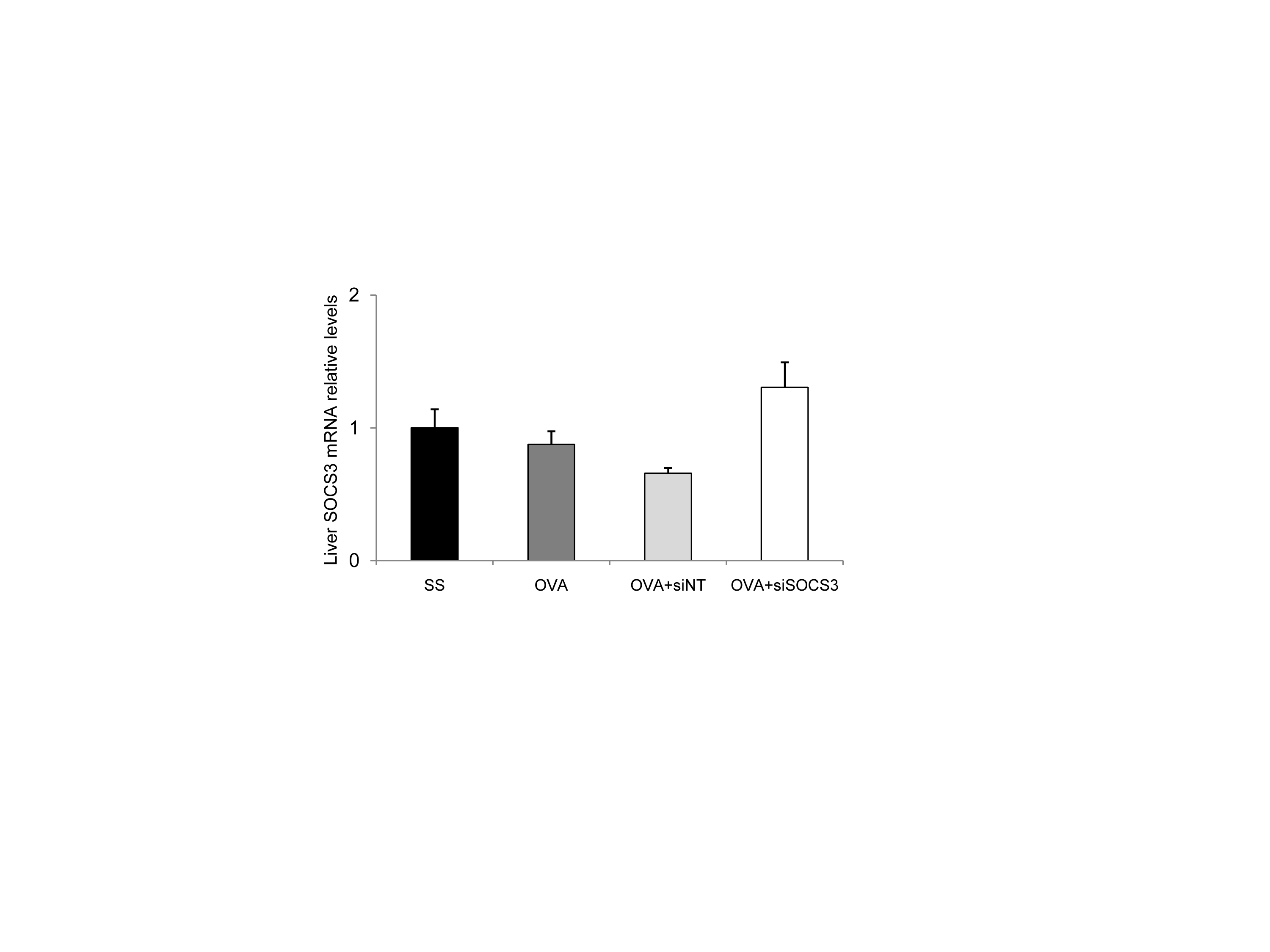

Supplement: Figure S2 — SOCS3 gene expression in liver after intranasal SOCS3-siRNA delivering. (TIF) [file pone.0091996.s002.tif]

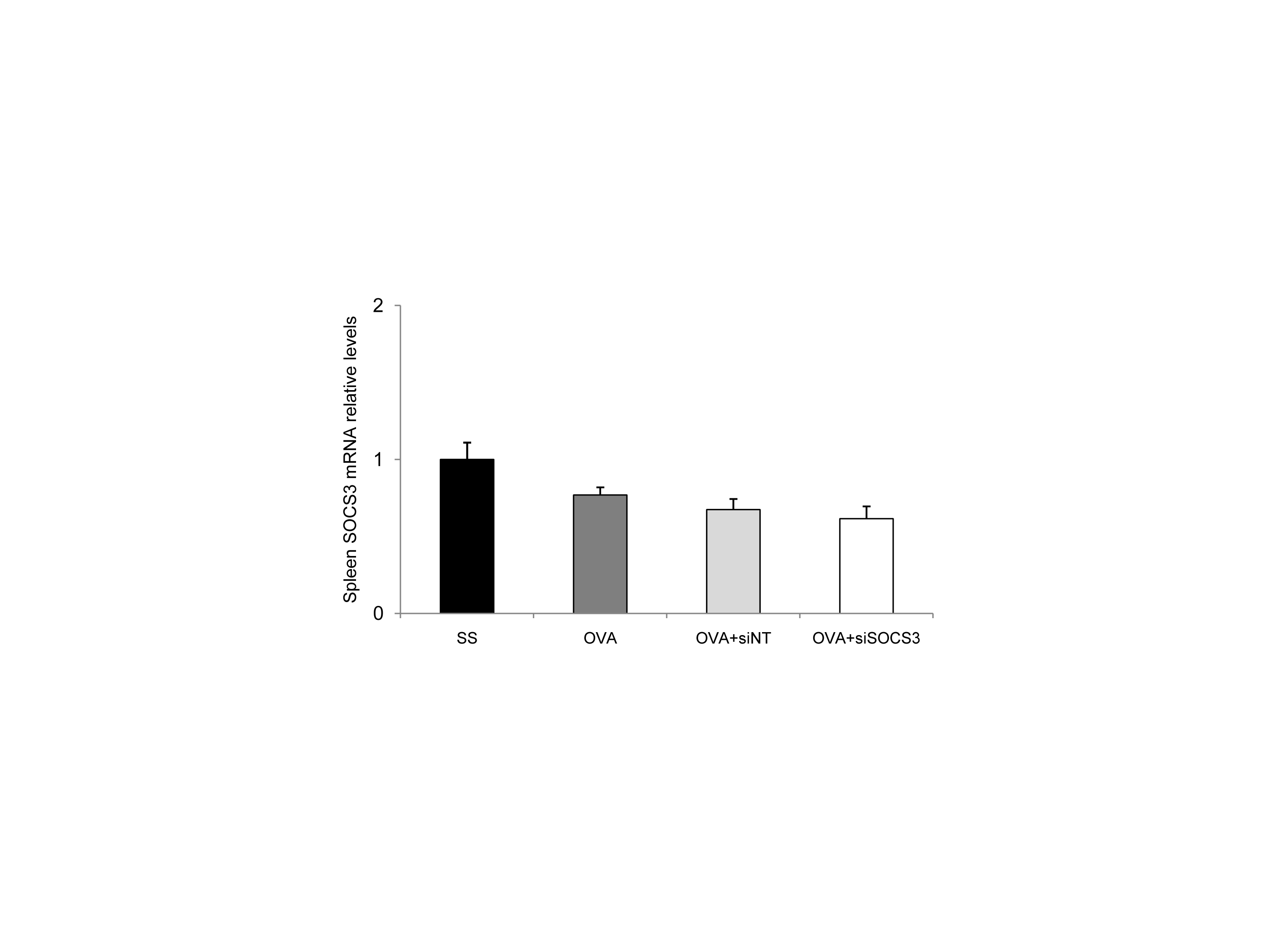

Supplement: Figure S3 — SOCS3 gene expression in spleen after intranasal SOCS3-siRNA delivering. (TIF) [file pone.0091996.s003.tif]

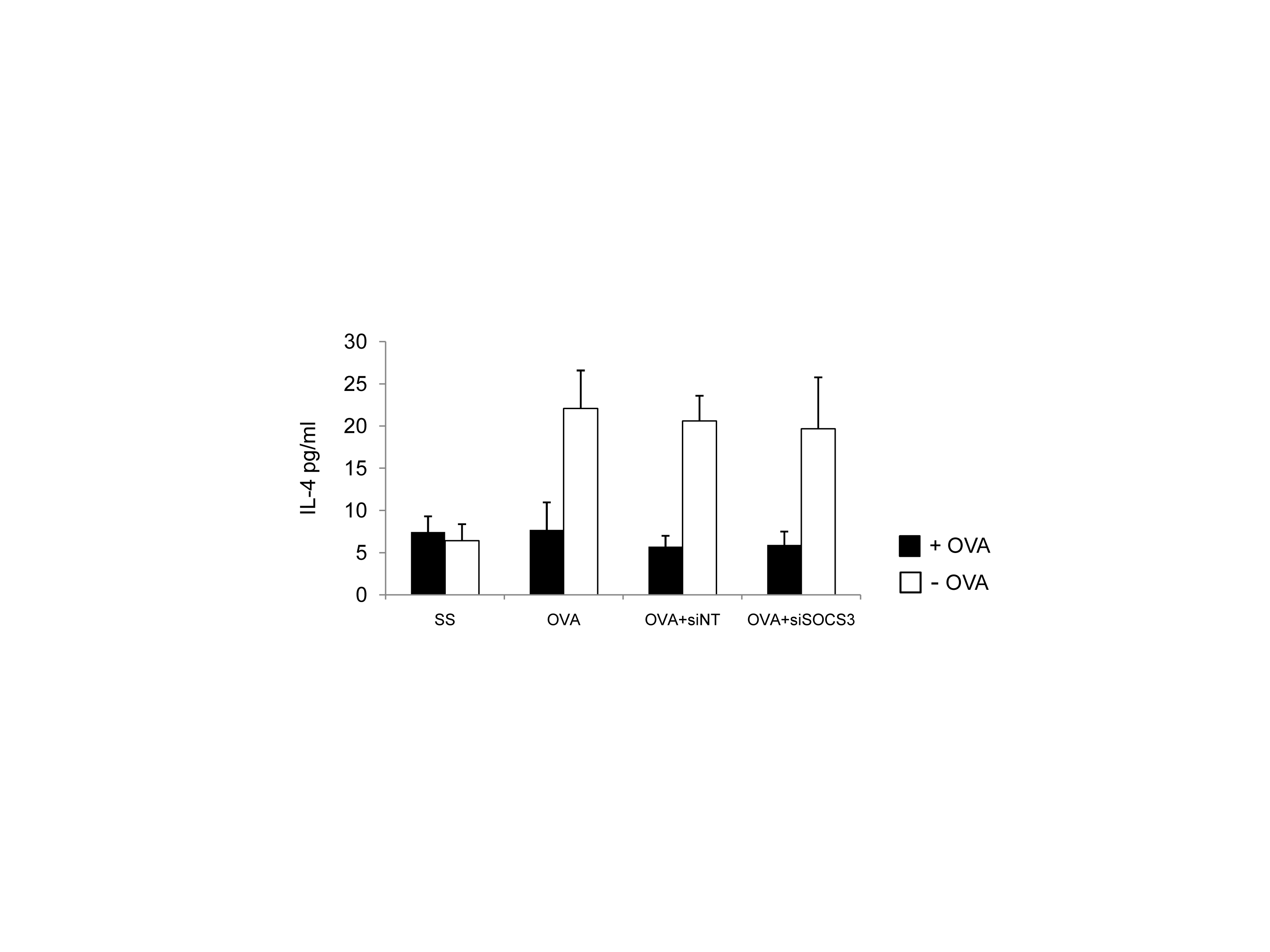

Supplement: Figure S4 — OVA treatment up regulates IL-4 expression in culture splenocytes from mice previously OVA immunized. (TIF) [file pone.0091996.s004.tif]
